# Impact of different 3D regions of interest on quantifying dynamic lumbar vertebral microstructure in ovariectomized rats—a micro-CT study

**DOI:** 10.3389/fmed.2024.1503761

**Published:** 2025-02-17

**Authors:** Huihui Xu, Hong Liu, Meijie Liu, Yan Li, Jinghua Pan, Shaojun Wang, Guowei Wang, Xin Liu, Ying Liu, Xiaoqin Hou, Hongyan Zhao

**Affiliations:** ^1^Department of Joints and Soft Tissue Injury, Shenzhen Traditional Chinese Medicine Hospital, The Fourth Clinical Medical College of Guangzhou University of Chinese Medicine, Shenzhen, China; ^2^Beijing Key Laboratory of Research of Chinese Medicine on Prevention and Treatment for Major Diseases, Experimental Research Center, China Academy of Chinese Medical Science, Beijing, China; ^3^Institute of Basic Theory of Chinese Medicine, China Academy of Chinese Medical Science, Beijing, China; ^4^Fangta Hospital of Traditional Chinese Medicine, Shanghai, China; ^5^Department of Clinic No. 1 Office, Shenzhen Traditional Chinese Medicine Hospital, The Fourth Clinical Medical College of Guangzhou University of Chinese Medicine, Shenzhen, China

**Keywords:** micro-CT, regions of interest, ovariectomy, lumbar vertebrae, osteoporosis

## Abstract

**Introduction:**

The selection of regions of interest (ROIs) is crucial for accurate microcomputed tomography (micro-CT) analysis. Distinct ROI selection methods exist for lumbar vertebras in osteoporotic animal model research. However, whether different ROIs directly affect the results of quantitative micro-CT-based microarchitectural data is still unknown. This study aimed to compare the diagnostic accuracy of two commonly used ROIs of lumbar vertebras in ovariectomized (OVX) rats at different time points.

**Methods:**

Rats were randomly divided into the baseline group, the sham/OVX-operated groups, with 12- or 24-weeks sham (Sham 12w or Sham 24w)/12- or 24-weeks (OVX 12w or OVX 24w)-operated group (*n* = 6 in every group). The fifth lumbar vertebras were collected and scanned using micro-CT. Quantitative analyses of bone microarchitecture parameters were conducted separately for the central ROI (ROI 1) and overall ROI (ROI 2).

**Results:**

The results indicated that the Tb.N of baseline group rats for ROI 1 was significantly lower than that for ROI 2. The Tb.Th of rats of the Sham 12w and Sham 24w groups was significantly increased compared to that of the baseline group rats using the ROI 2 analysis. The bone mineral density (BMD) and bone volume fraction (BV/TV) were significantly lower by the ROI 1 than by the ROI 2 in all groups. The BMD and BV/TV also showed a significant reduction at 24 weeks postoperatively compared with those at 12 weeks postoperatively. Bland–Altman analysis showed good consistency between the two different ROI selection methods.

**Conclusion:**

This study found that capturing peripheral trabeculas (overall ROI) does not explain the increased Tb.Th in healthy mice and decreased Tb.N in OVX mice; both findings indicate that this is evident in both ROI. Moreover, this study suggested the potential value of the central ROI (effective and quicker) for evaluating osteoporosis of the lumbar vertebras in OVX rats and provides a basis for analyzing the morphological changes of lumbar trabecular.

## 1 Introduction

Osteoporosis is a bone metabolism disease characterized by bone mass reduction and bone tissue microstructural degeneration. Vertebral trabecular bone is microstructurally heterogeneous; the distribution and variation of microstructural parameters in vertebral trabeculas change with age (1, 2). Clinically, assessment of lumbar vertebras by computerized tomography has commonly been used for the early diagnosis of osteoporosis (3). A clinical human study reported that the attenuation value of the fourth lumbar trabecular ROI is the most relevant measurement for predicting osteoporotic compression fractures (4). In fundamental research, microcomputed tomography (micro-CT) is a powerful tool for evaluating bone properties and microstructure (5); It shows the potential to predict structural failure of the lumbar spine, thereby aiding the diagnosis of osteoporosis and assessment of its treatment (6). Based on the measurement of geometrical and densitometric properties, this method is recognized as the standard reference for evaluating bone health and has frequently been used in many studies (7–11). Its derived imaging utilized for three-dimensional (3D) reconstruction of morphological evaluations can accurately depict bone microstructural parameters for quantitative assessments. Additionally, micro-CT could provide reproducible assessment for various skeletal regions of multiple species, such as the tibia, femur, craniofacial skeleton, vertebras, and mandibular bone microarchitecture (12–14). However, whether region-of-interest (ROI) selection is accurate and reliable directly affects the results of quantitative micro-CT analysis. Thus, the different ROI selection methods can influence the micro-CT-based microarchitectural data.

In recent studies, various ROI extraction approaches have been utilized in the microarchitecture analysis of different types of bone (15, 16). There are also distinct ROI selection methods for lumbar vertebras in current osteoporotic animal model research (17–20). One standard ROI selection method is drawing an ROI including the overall lumbar cancellous vertebral body region (overall ROI) and excluding the cortical bone (21). The performance of the overall ROI method analysis is a well-recognized but relatively more complicated method since it can analyze all bone trabeculas of the species. Drawing an ROI including same-size cylinder contours in the central lumbar vertebral body region (central ROI) excluding the cortical bone is another method (22). The performance of the central ROI method analysis is relatively more convenient, but only the central bone trabeculas can be analyzed, and its accuracy is unclear.

Bilateral ovariectomy (OVX) rat is generally used as a classical osteoporosis animal model (23). Using micro-CT, the major predictors for assessing bone degeneration in animal osteoporosis models include bone mineral density (BMD) and other microstructural parameters such as the percentage bone volume fraction (BV/TV), trabecular bone thickness (Tb.Th), trabecular bone number (Tb.N), trabecular bone spacing (Tb.Sp), and trabecular bone structural model index (SMI) based on bone microarchitecture. Therefore, based on ex vivo micro-CT images, this study explored the accuracy of central ROI compared with the overall ROI method for evaluating lumbar vertebral bone mass and microstructure in OVX rats at different time points after ovariectomy.

## 2 Materials and methods

### 2.1 Experimental animals and study design

Thirty 11-week-old Sprague–Dawley (SD) female rats were purchased from Beijing Vital River Laboratory Animal Technology Co., Ltd (Beijing, China). Before the start of the experiment, all rats were allowed to acclimatize for 1 week. They were given ad libitum access to a standard rodent chow diet and regular drinking water. They were maintained under specified pathogen-free (SPF) conditions with a cycle of 12 h light/12 h darkness, a temperature of 22 ± 2°C, and a 60% relative humidity. Then, rats were randomly divided into the baseline group, which were sacrificed by cervical dislocation under euthanasia (pentobarbital sodium, 45 mg/kg, i.p.) without prior interventions on the day of surgery. The remainder were assigned to either the sham-or OVX-operated groups, with the 12 weeks sham-operated group (Sham 12w), 24 weeks sham-operated group (Sham 24w), 12 weeks OVX group (OVX 12w), and 24 weeks OVX group (OVX 24w) (*n* = 6 in every group).

### 2.2 Surgical procedure

Rats in the OVX group were anesthetized by intraperitoneal injection of pentobarbital sodium (45 mg/kg, i.p.) and subjected to bilateral ovariectomy to induce bone loss. Briefly, a dorsal incision was made around the midpoint between the last rib and iliac crest; subsequently, rats were subjected to bilateral ligation and removal of ovaries in the OVX group. The sham group underwent the same surgery with retained ovaries, but the same quantity of retroperitoneal adipose tissue alongside the preserved bilateral ovaries were resected. To prevent surgical infection, penicillin was used routinely. After surgery, all rats were maintained in a warm environment. Moreover, rats in the baseline group were sacrificed by cervical dislocation under euthanasia (pentobarbital sodium, 45 mg/kg, i.p.) to obtain basal vertebral data based on micro-CT. The rats in sham-or OVX-operated groups were, respectively, sacrificed by cervical dislocation at 12 weeks or 24 weeks postoperatively after being anesthetized with intravenous pentobarbital sodium (45 mg/kg, i.p.). Subsequently, fifth lumbar vertebras (L5) specimens were collected to analyze the vertebral trabecular bone mass and microstructure. L5 were dissected from the animals immediately after sacrifice and washed with phosphate-buffered saline after removing the connected muscle and soft tissues. The vertebra specimens were subsequently fixed with 4% paraformaldehyde.

### 2.3 Micro-CT analysis and the ROI setting

The lumbar vertebra was mounted on moist paper and scanned [Skyscan, 1,276 (Saarbrücken, SL, Germany); Bruker MicroCT, Belgium (Saarbrücken, SL, Germany)] at 10.2-μm nominal resolution, with an X-ray tube voltage of 70 kV, current of 200 μA, and a 0.5 mm aluminum filter. The scanning angular rotation was 180°, and the angular increment was 0.60°. NRecon (version 1.6.9.8, Bruker MicroCT) performed reconstruction after scans. In addition, a threshold of 95–255 was used.

Then, the ROI of trabecular bone was selected, respectively, based on two methods. The central ROI selection method (ROI 1, a convenient method, but accuracy is unclear) involved drawing cylinder contours (diameter, 2 mm) in the central lumbar vertebral body region with the aid of CT software, while the other method (ROI 2, a well-recognized but complicated method) was identified by drawing the overall lumbar cancellous vertebral body region. Furthermore, both methods excluded the cortical bone when selecting the ROI. The trabecular bone was analyzed 4.2 mm below the proximal endplate in both methods.

**Figure 1:**
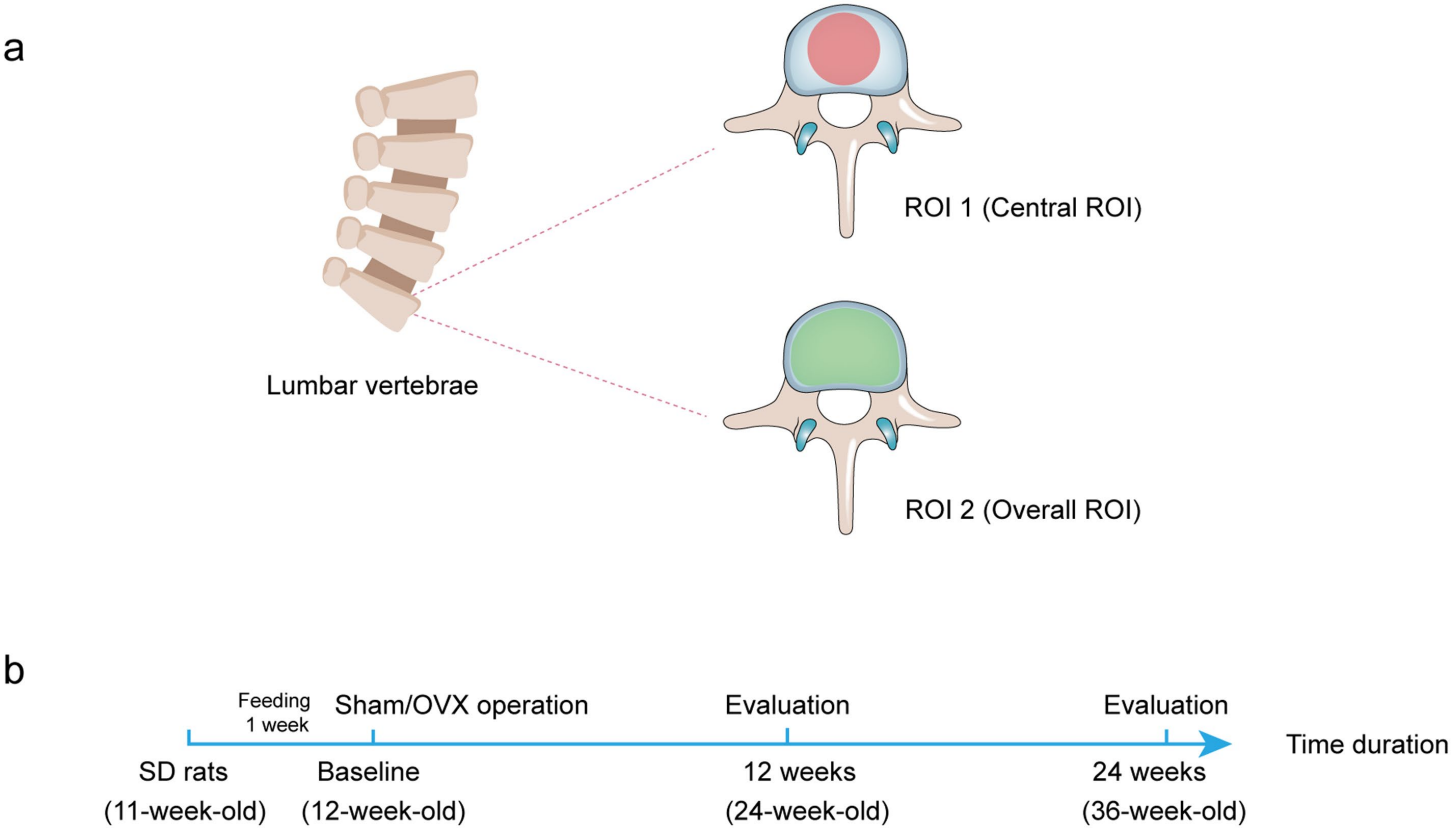
Diagram of two different ROI selection. **(A)** Transverse plane of rat lumbar spine to display the ROI shape. The ROI is drawn in the bone trabecular volume with a red area (central ROI) or green area (overall ROI). **(B)** The treatment protocol for the *in vivo* study.

The data were analyzed using CTAn software (version 1.13.2.1, Bruker MicroCT) (Saarbrücken, SL, Germany). Then, the following parameters according to the guidelines of the ASBMR nomenclature committee (24) were detected: BMD (g/cm^3^), bone volume to tissue volume fraction (BV/TV, %), trabecular number (Tb.N, 1/mm), trabecular thickness (Tb.Th, mm), trabecular separation (Tb.Sp, mm), and structural model index (SMI) (Figure 1).

### 2.4 Statistical analysis

The statistical analyses were performed using the Statistical Package for the Social Sciences (SPSS) version 24.0 software program (Chicago, CA, United States). Results were presented as the mean ± SD. The normality test was applied to examine the normal distribution of data. If data followed a normal distribution, *t*-tests were used to compare the two groups. When the data were not normally distributed, the Mann–Whitney U test was used to compare the two groups. The *p*-value <0.05 was considered to reflect a statistically significant difference for all experiments. Bland–Altman plots were used to describe agreement between two quantitative measurements, including the 95% confidence interval (CI) of the lower and upper limits of agreement (LoA). The averages of the two quantitative measurements were calculated and then plotted against their difference. The normality distribution of the differences was assessed, and the mean difference and LoA were drawn. Horizontal lines were drawn at the mean difference and LoA, defined as the mean difference ± 1.96 times the SD of the differences.

## 3 Results

### 3.1 Time-course variation of trabecular bone morphologic parameters in the lumbar vertebras of sham-operated rats

**Figure 2:**
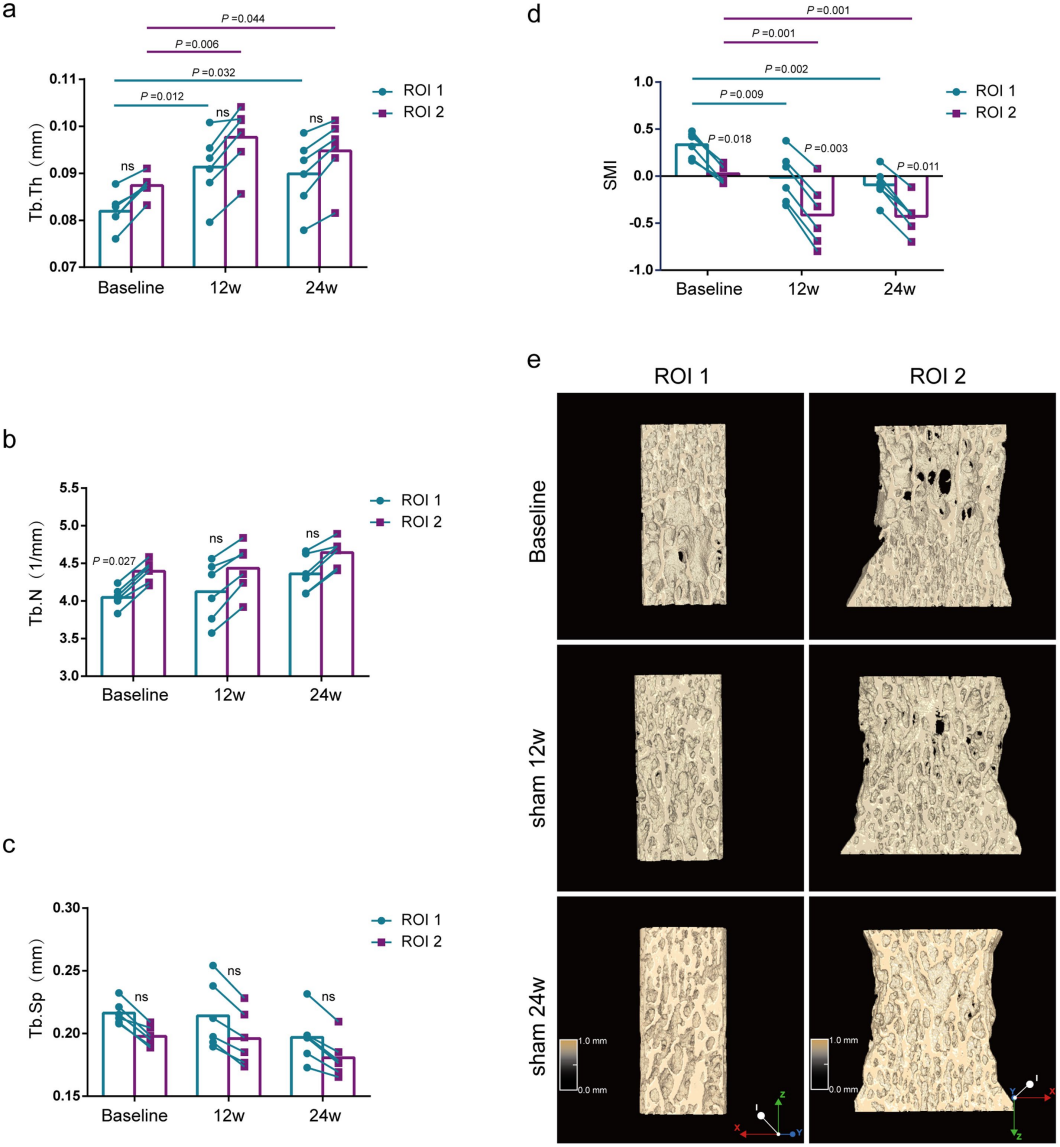
Evaluation of lumbar trabecular bone microstructural changes in sham rats at different age by two ROI selection methods. **(A)** Tb. Th; **(B)** Tb. N; **(C)** Tb. Sp; **(D)** SMI; **(E)** Representative 3D reconstruction and longitudinal sectional images of L5 by micro-CT (*n* = 6). ns: no significant.

Comparison of bone morphological parameters between two methods at the same time point. As shown in Figure 2, the bone morphologic parameters (Tb.Th and Tb.Sp) were not statistically different between the two methods in the baseline group (rat at 12 weeks), Sham 12w group (rat at 24 weeks), and Sham 24w group (rat at 36 weeks) (Figures 2A,C). The Tb.N of the baseline group by the ROI 1 was significantly lower than that by the ROI 2 (*p* = 0.027) (Figure 2B). Although the 24-and 36-week rats also tended to exhibit lower Tb.N by ROI 1 compared with those by ROI 2, no significant difference was found between the two methods (Figure 2B).

Comparison of bone morphological parameters at different time points. Using the ROI 2 analysis, the Tb.Th of rats of the Sham 12w and Sham 24w groups was significantly increased compared with that of the baseline group rats (*p =* 0.044, *p =* 0.006), whereas rats of the Sham 12w and Sham 24w groups showed no significant difference between each other by the ROI 2 (Figure 2A). Additionally, no significant differences were found in Tb.N and Tb.Sp in all different time groups (Figures 2B,C). SMI of the Sham 12w and Sham 24w groups indicated quality improvement of bone tissue compared with the baseline group (*p* = 0.001) (Figure 2D). Furthermore, we found that the results of the ROI 1 analysis were consistent with the ROI 2 analysis when using the same analytical parameters (Figures 2A–C).

### 3.2 Time-course variation of trabecular bone mass parameters in the lumbar vertebras of sham-operated rats

**Figure 3:**
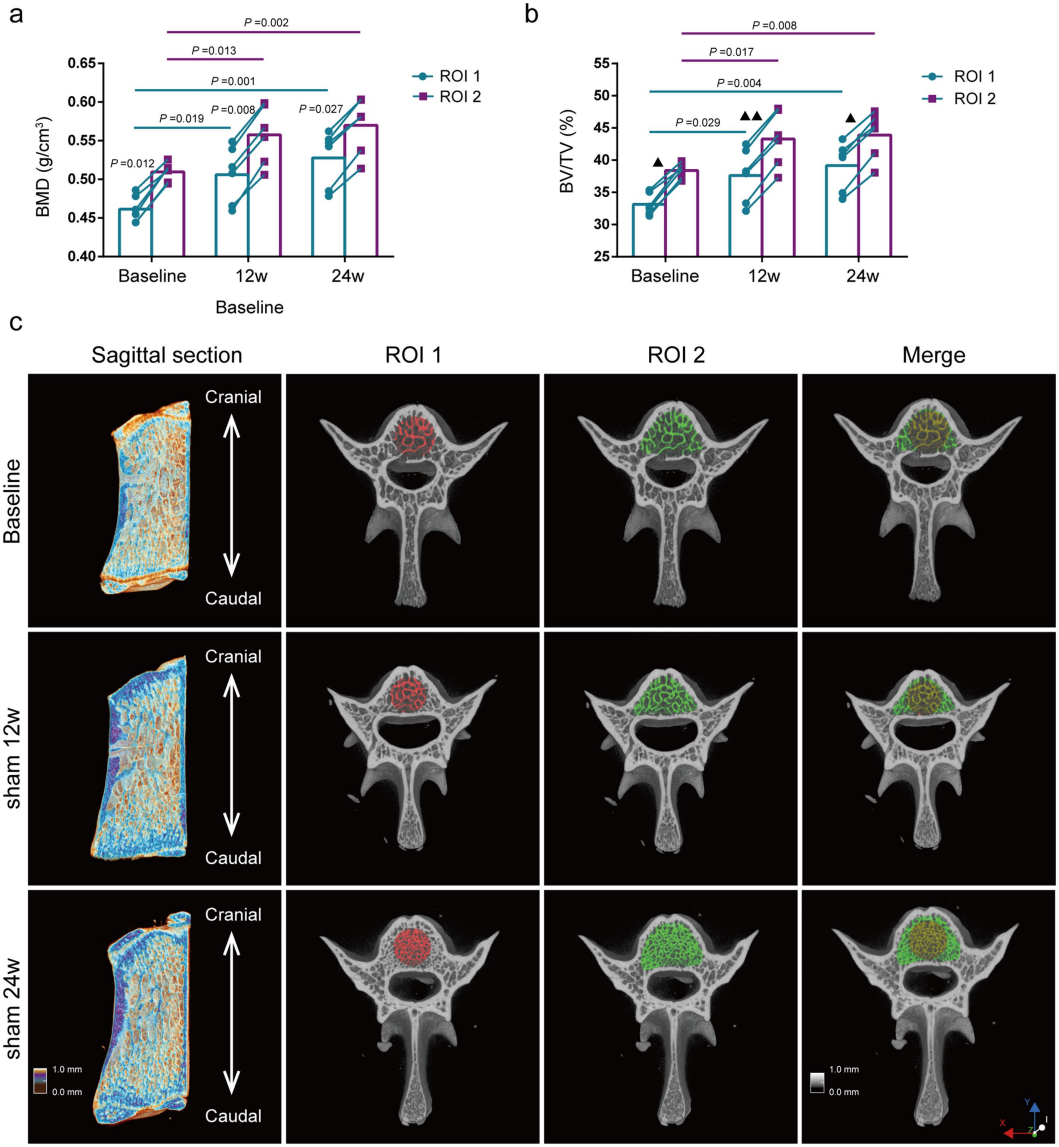
Evaluation of lumbar trabecular bone mass changes in sham rats at different age by two ROI selection methods. **(A)** BMD; **(B)** BV/TV; **(C)** Representative 3D reconstruction of sagittal and cross-sectional images of L5 by micro-CT, the ROI is displayed with the red area (central ROI), green area (overall ROI), and yellow area (merge) (*n* = 6). ns: no significant.

Comparison of bone mass parameters between two methods at the same time point. The BMD of the lumbar vertebras was significantly lower by the ROI 1 than by the ROI 2 at all different ages of rats (*p* = 0.012, *p =* 0.008, *p* = 0.027) (Figure 3A), and the results of BV/TV were also consistent with the BMD results (Figure 3B).

Comparison of bone mass parameters at different time points. The BMD of the lumbar vertebras in rats of the Sham 12w and Sham 24w groups were significantly increased compared with the baseline group by the ROI 2 (*p* = 0.013, *p* = 0.002,). Still, there was no significant difference between the rats of the Sham 12w and Sham 24w groups (Figure 3A). Moreover, the changes in BV/TV were consistent with those in BMD (Figure 3B). Thus, we presumed that the increase of BMD and BV/TV in adult rats over time was mainly due to the rise of trabecular thickness. Furthermore, we found that the statistical results for BMD and BV/TV by the ROI 1 were consistent with the results of the ROI 2 (Figures 3A–C).

### 3.3 Time-course variation of trabecular bone morphologic parameters in the lumbar vertebras of OVX rats

Subsequently, we compared the alteration of trabecular bone morphologic parameters in the lumbar vertebras of OVX rats of the 12 and 24 weeks after surgery. Ovariectomy induced bone loss in the lumbar vertebral trabecular bone, which was increased over time in severity (Supplementary Figure S1).

**Figure 4:**
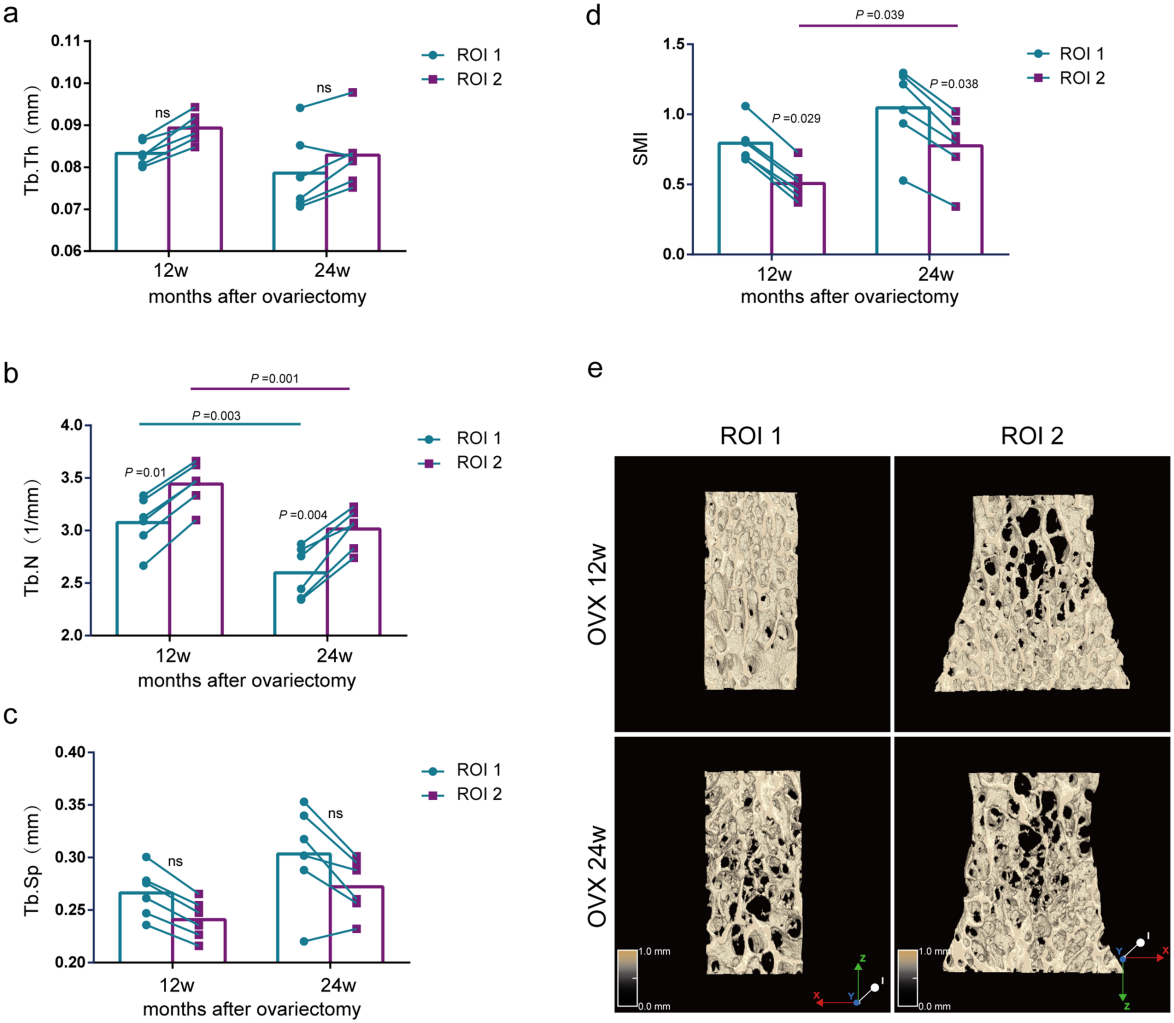
Evaluation of lumbar trabecular bone microstructural changes in OVX rats at different times after surgery by two ROI selection methods. **(A)** Tb. Th; **(B)** Tb. N; **(C)** Tb. Sp; **(D)** SMI; **(E)** Representative 3D reconstruction and longitudinal sectional images of L5 by micro-CT (*n* = 6). ns: no significant.

Comparison of bone morphological parameters between two methods at the same time point. As shown in Figure 4, although there was no significant difference in Tb.Th or Tb.Sp by the ROI 1 compared with the ROI 2 at 12 and 24 weeks after ovariectomy surgery (Figures 4A,C), the Tb.N of the lumbar vertebras was significantly lower by ROI 1 compared to ROI 2 (*p* = 0.01, *p* = 0.004) (Figure 4B).

Comparison of bone morphological parameters at different time points. The ROI 2 analysis showed that the Tb.N of the lumbar spine was significantly reduced in the 24-week postoperative rats compared with the 12-week postoperative rats (*p* = 0.001) (Figure 4B), but there was no significant difference in Tb.Th and Tb.Sp (Figures 4A,C). Using the same analytical parameters and selecting the central region for analysis, we found that the results of the ROI 1 were consistent with those of the ROI 2 (Figures 4A–C). Notably, the OVX 24w group presented a significantly higher SMI index than the OVX 12w group by the ROI 1, indicating less parallel trabeculas (*p* = 0.039) (Figure 4D). Although the OVX 24w group showed a higher SMI index than the OVX 12w group by the ROI 2, there was no significant difference.

### 3.4 Time-course variation of trabecular bone mass parameters in the lumbar vertebras of OVX rats

**Figure 5:**
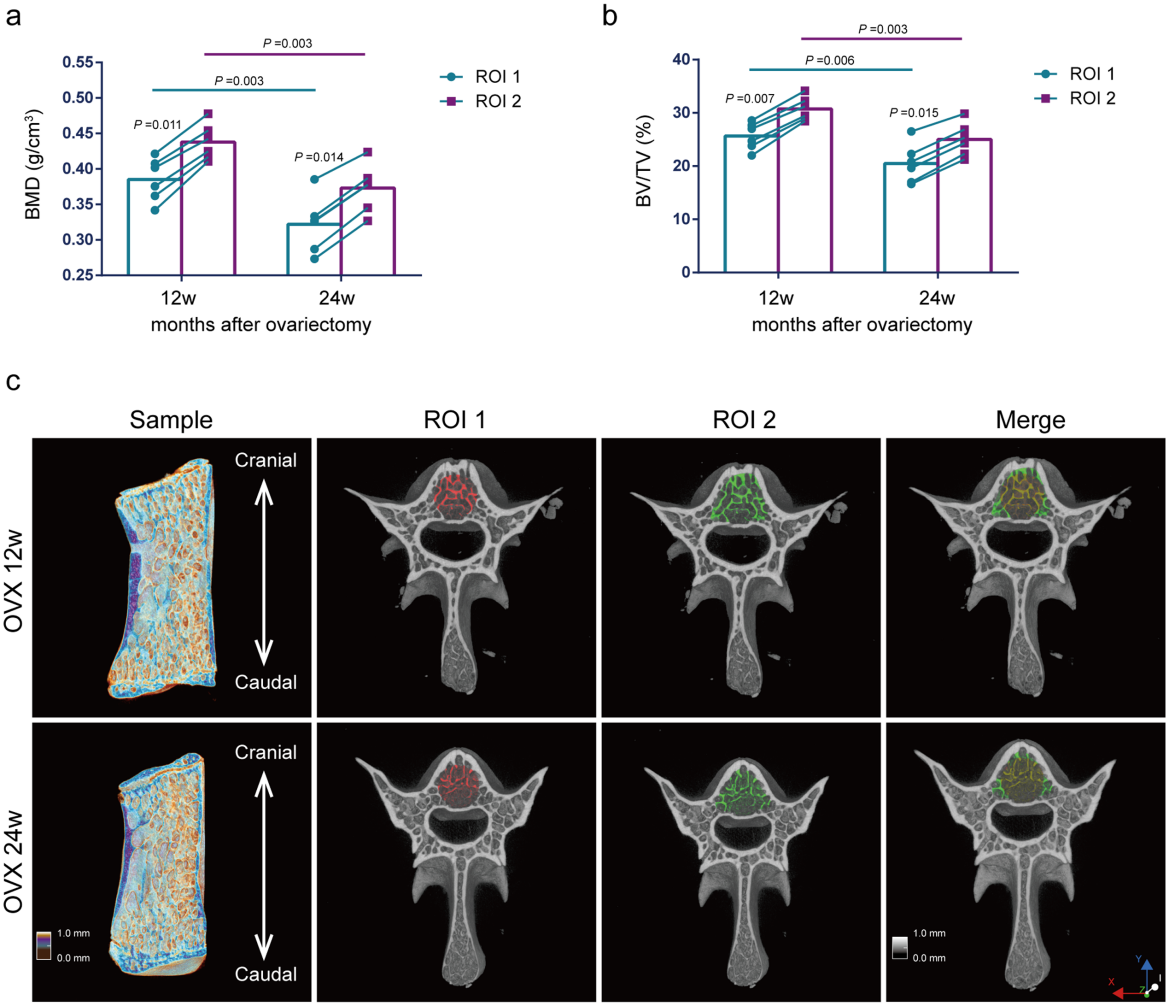
Evaluation of lumbar trabecular bone mass changes in OVX rats at different times after surgery by two ROI selection methods. **(A)** BMD; **(B)** BV/TV; **(C)** Representative 3D reconstruction of sagittal and cross-sectional images of L5 by micro-CT. The ROI is displayed with the red area (central ROI), green area (overall ROI), and yellow area (merge) (*n* = 6). ns: no significant.

Comparison of bone mass parameters between two methods at the same time point. As shown in Figure 5, at 12 weeks and 24 weeks after ovariectomy surgery, BMD and BV/TV using the ROI 1 were significantly lower compared with the ROI 2 (*p* = 0.011, *p* = 0.014, *p* = 0.007, *p* = 0.015) (Figures 5A,B).

Comparison of bone mass parameters at different time points. The analysis of ROI 2 methods showed that the BMD and BV/TV were significantly reduced at 24 weeks postoperatively compared with those at 12 weeks postoperatively (*p* = 0.003, *p* = 0.003) (Figures 5A,B). Thus, we presumed that the decreases in BMD and BV/TV of osteoporosis rats induced by ovariectomy surgery were mainly due to the decreased Tb.N. The results using the ROI 1 were consistent with those using the ROI 2 for the analysis of BMD and BV/TV (Figures 5A,B).

### 3.5 Bland–Altman analysis showed good consistency between the two different ROI selection methods

**Figure 6:**
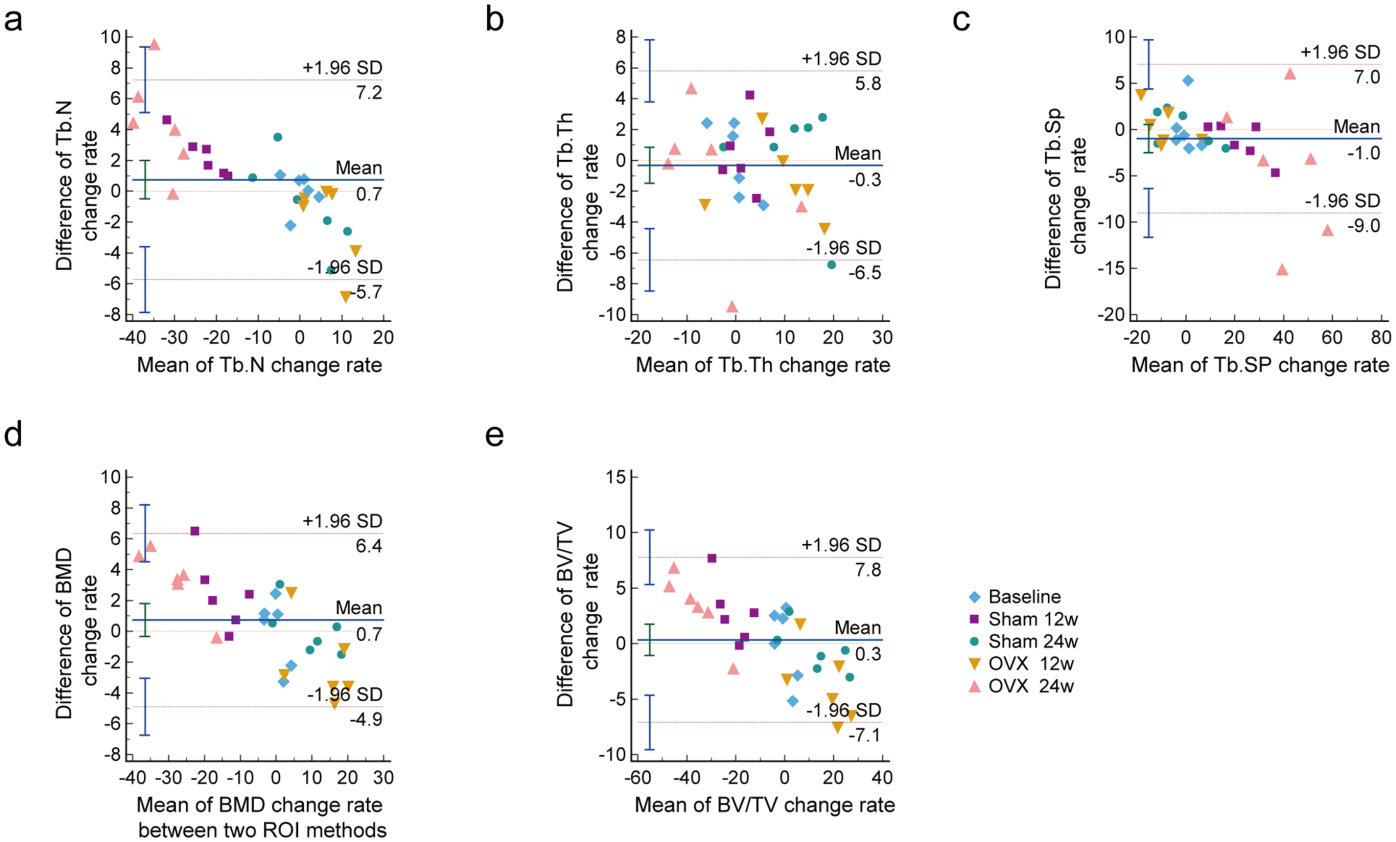
Bland-Altman plots of the change rate of lumbar vertebral microarchitecture skeletal parameters with a 95% CI. **(A)** Tb.N change rate; **(B)** Tb.Th change rate; **(C)** Tb.Sp change rate; **(D)** BMD change rate; **(E)** BV/TV change rate. It includes the mean difference (blue solid line) and the limits of agreement (red dotted line) with an approximate 95% CI (*n* = 6). CI confidence interval, Tb. N Trabecular bone number, Tb. Th Trabecular bone thickness, Tb. Sp Trabecular bone spacing, SMI Trabecular bone structural model index.

Bland–Altman’s analysis is commonly used to compare two measurement methods, primarily to assess the bias and precision of a new measuring method (25). Bland–Altman plots with the approximate CI of the LoA are displayed in Figure 6. The *x*-axis shows the mean change rate of Tb. N, Tb.Th, Tb.SP, BMD, and BV/TV between the central and overall ROI methods. The *y*-axis was plotted against the differences in the change rate of Tb.N, Tb.Th, Tb.SP, BMD, and BV/TV by the central ROI and overall ROI methods. The change rate of Tb.N was calculated using the following formula (same as for the other parameters):


[Tb.N(actual data)−Tb.N(baseline)]/Tb.N(baseline)

As shown in Figure 6, the difference between the two methods was within the consistency limits, which was acceptable and denoted as good consistency. The relative change rates for all parameters in all groups are shown relative to basic data with a 95% CI.

## 4 Discussion

Vertebral trabeculas are microstructurally heterogeneous, and for lumbar vertebral trabeculas, the regional variations in the microstructural properties are crucial for diagnosis and treatment. They may help us gain more insight into the mechanism of vertebral osteoporosis and the related fracture risks. Amélie Poilliot examined the subchondral bone density distribution of the superior and inferior endplates with micro-CT scans and determined cancellous and compartment-specific trabecular architecture in the cervical vertebra to aid with the successful integration of orthopedic implants. (The quantification of the 3D-trabecular architecture of the fourth cervical vertebra using CT osteoabsorptiometry and micro-CT). The vertebral trabecular bone is one of the primary sites for bone loss. He Gong and H. Chen divide the lumbar spine into the anterior, posterior, central, right, and left regions. They found that significant differences between women and men are observed at some microstructural parameters, and age-related vertebral trabecular bone loss may be caused by increased resorption activity. These findings illustrate potential mechanisms underlying vertebral fractures (regional variations of vertebral trabecular bone microstructure with age and gender) (Regional variations in microstructural properties of vertebral trabeculas with aging). In this study, we explored the accuracy of central ROI compared with the overall ROI method for evaluating the density and skeletal microarchitectural parameters of lumbar vertebral trabecular bone in OVX rats at different time points using micro-CT analysis. Our study discovered that when the sham rats reached 24 weeks old, the bone morphology which became stable, so did the bone mass. Bone growth is an essential part of skeletal development during youth, and characterizing bone growth helps to better understand the determining factors of peak bone mass. A previous study reported that the micro-CT-based method could serve as an alternative non-destructive method for the quantification of longitudinal bone growth rates of young SD rats with highly reproducible measurements (5). The SMI values near zero present the bone trabeculas as parallel plates, promoting higher bone density. Our results related to SMI of the 24- and 36-week old rats indicated improvement of quality of bone tissue compared with the baseline group.

Micro-CT is a common, non-destructive, and reliable method that can detect bone microarchitectural alterations in physiological and pathological conditions in animal models, including OVX rats (26–28). This study’s analyses of lumbar vertebras by the ROI 2 method revealed markedly decreased BMD, BV/TV, Tb.Th, and Tb.N and significantly increased Tb.Sp in the OVX group at two distinct time points compared with rats of the same age in the sham-operated group. Interestingly, the ROI 2 analysis showed that the bone microarchitectural alteration by ovarian surgery is mainly due to decreased Tb.N. Analysis of the two methods also showed that the BMD and BV/TV were significantly reduced at 24 weeks compared to 12 weeks postoperatively.

Micro-CT can potentially be applied to predict the location of structural failure in animal models of osteoporosis. In postmenopausal osteoporosis, decreasing estrogen levels result in substantial bone loss and increased fracture risk. Vertebral bodies have a relatively high proportion of trabecular bone, and osteoporotic fractures occur much earlier in the lumbar spine than in the cervical vertebras in osteoporosis (29). Hormonal reduction induced in female rats (ovariectomy surgery) played a role in the imbalance in bone remodeling. Previous studies reported that a 3-month period after OVX surgery was sufficient to induce bone loss and significantly lower bone quantity in rats (30). Bone microarchitecture is usually performed on the vertebras in rats, and bone metabolism in the lumbar vertebras is less active than that in other skeletal sites (31). Ozasa et al. assessed the L5 vertebral cortex along the craniocaudal axis of the vertebra for OVX rats to demonstrate the changes in volumetric BMD and bone mechanical properties due to osteoporosis (32). Muller et al. found that the macroscopic vertebras stiffness and the microscopic modulus of OVX rats diminish by approximately 70% with the progression of osteoporosis at the age of 12 and 14 months (33). As shown in Supplementary Figure S1, BMD, BV/TV, Tb.Th, and Tb.N were significantly lower, and the Tb.Sp was considerably higher in the OVX 12w and OVX 24w group compared to the sham-operated group of the same duration.

The application of micro-CT on bone health in different small animal studies has exponentially risen in recent years, and there are extensive studies using micro-CT to study various animal models of bone microarchitectural changes (34, 35). Zhu et al. used the central ROI method to observe the L5 morphological reconstruction of vertebral bone at baseline, 4, 8, and 12 months in OVX rats after surgery through micro-CT. They found that the trabecular gaps were significantly expanded, and trabecular fracture and perforation significantly increased (36). Guo et al. used the overall ROI method manually to prove that bone loss patterns of the L5 vertebral body and femur were distinct in the early stage of estrogen deficiency (37). Our study showed that sham rats’ increased BMD and BV/TV were mainly due to the increased Tb.Th. Moreover, we presumed that the decreases in BMD and BV/TV of OVX rats were mainly due to the decreased Tb.N. Thus, when combined with the morphological parameters, we discovered that in addition to the higher Tb.N, the trabeculas in the periphery of the lumbar spine were denser than those in the center of the trabeculas. In this study, the results using the ROI 1 were consistent with those of the overall ROI methods when the same parameters were used in the analysis.

In addition, we used the Bland–Altman agreement analysis to evaluate the agreement between the two methods. The Bland–Altman analysis is the gold standard for analysis involving statistical agreement. The main principle of the limits of agreement in the Bland–Altman approach is to measure the difference of observations between two methods (38). The Bland–Altman’s analysis is commonly used to assess the bias and precision of measuring methods. Moreover, the difference between ROI 1 and ROI 2 in both the sham and OVX groups was within the consistency limits, which was acceptable and denoted as good consistency. However, there are also some limitations in this research. The two methods are largely consistent with detecting loss of trabecular number, thickness, BV/TV, or BMD, and were validated in both healthy and ovariectomized (osteoporotic) mice. The nature of the larger ROI also the overall method will always capture more bone due to incorporating the peripheral trabeculas. Nevertheless, the central ROI may be just as effective (and quicker) in measuring differences in lumbar vertebras. Moreover, this study only observed the dynamic changes of bone trabeculas in healthy rats and OVX rats by the two ROI, and did not explore them with other scenarios/diseases/models, which is a limitation of this research. In addition, standardizing the central ROI is not easy to achieve. The height and diameter of the central ROI should vary if the animals differ in size with normal biological variability or age.

## 5 Conclusion

This study observed that the capturing of peripheral trabeculas (overall ROI) does not account for the increased Tb.Th in healthy mice and decreased Tb.N in OVX mice; both significances indicate that this is detected in both central and overall ROI, and so is instead related to the healthy mice (where trabecular thicken with age) and OVX mice (where trabeculas are lost with osteoporosis). Moreover, this study demonstrates the potential value of the central ROI for evaluating osteoporosis of the lumbar vertebras in OVX rats and provides a basis for analyzing the morphological changes of lumbar trabeculas.

## Data Availability

The raw data supporting the conclusions of this article will be made available by the authors, without undue reservation.
