# Supplementary material for: Impact of different 3D regions of interest on quantifying dynamic lumbar vertebral microstructure in ovariectomized rats—a micro-CT study
Source: Front Med (Lausanne). 2025 Feb 17;11:1503761. doi: 10.3389/fmed.2024.1503761 (PMC11873078; doi:10.3389/fmed.2024.1503761)
Supplement: Supplementary file 1 [file Table_1.doc]

**Supplementary Materials**

**Impact of different 3D regions of interest on quantifying dynamic** **lumbar vertebral microstructure in ovariectomized rats: a** **micro-CT study**

Huihui Xu^1,2#^, Hong Liu^2#^, Meijie Liu^2#^, Yan Li^2^, Jinghua Pan^2^, Shaojun Wang^2^, Guowei Wang^3^, Xin Liu^2^, Ying Liu^4^*, Xiaoqin Hou^5^* and Hongyan Zhao^2^*

*^1^ Department of Joints and Soft Tissue Injury, Shenzhen Traditional Chinese Medicine Hospital, The Fourth Clinical Medical College of Guangzhou University of Chinese Medicine, Shenzhen, Guangdong, 518033, China.*

*^2^Beijing Key Laboratory of Research of Chinese Medicine on Prevention and Treatment for Major Diseases, Experimental Research Center, China Academy of Chinese Medical Science, Beijing, 100700, China*

*^3^Institute of Basic Theory of Chinese Medicine, China Academy of Chinese Medical Science, Beijing, 100700, China*

*^4^Fangta Hospital of Traditional Chinese Medicine, Songjiang District, Shanghai, 201611, China*

*^5^ Department of Clinic No.1 office, Shenzhen Traditional Chinese Medicine Hospital, The Fourth Clinical Medical College of Guangzhou University of Chinese Medicine, Shenzhen, Guangdong, 518033, China.*

^#^ Huihui Xu, Hong Liu and Meijie Liu contributed equally to this study.

**^*^Correspondence:**

Ying Liu, E-mail: DORLIU20132023@163.com

Xiaoqin Hou, E-mail: 2444011989@qq.com

Hongyan Zhao, E-mail: zhaohongyan1997@163.com

**Differences in the ROI 1 and ROI 2 Methods Between OVX Rats and Sham-operated Rats at the Same Age**

As shown in supplementary Figure, using the ROI 2 analysis, the BMD, BV/TV, Tb. Th and Tb. N were significantly lower (*p*<0.05, *p*<0.01), and the Tb. Sp was significantly higher in the OVX group at two distinct time points (*P*<0.01) than in the same-month-old sham-operated group. The statistical results using the ROI 1 were consistent with the ROI 2 when choosing the same parameters.

**SUPPLEMENTARY FIGURE**

**
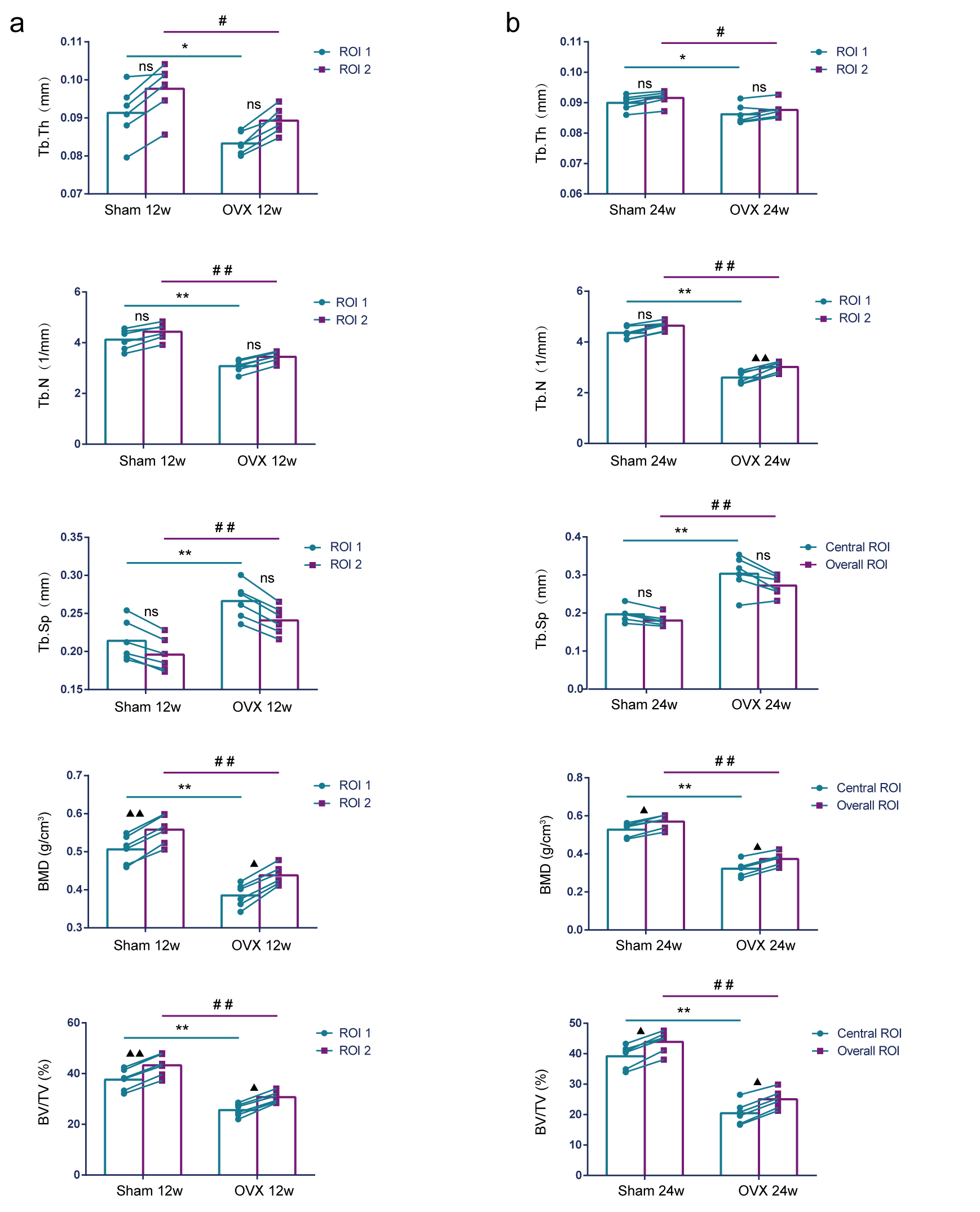
**

**Figure. S.** Comparison of the skeletal parameters of the lumbar vertebrae between OVX rats and sham-operated rats at different times after surgery by two ROI selection methods. (a) Bone morphologic and bone mass parameters at 12 weeks; (b) Bone morphologic and bone mass parameters at 24 weeks (*n=6*). **p*<0.05, ***p*<0.01, OVX group compared with Sham-operated group by central ROI method; # *p* <0.05, ## *p* <0.01, OVX group compared with Sham-operated group by overall ROI method; ▲*p* <0.05, ▲▲*p* <0.01, the ROI 1 compared with the ROI 2.
